# WWOX controls hepatic HIF1α to suppress hepatocyte proliferation and neoplasia

**DOI:** 10.1038/s41419-018-0510-4

**Published:** 2018-05-03

**Authors:** Muhannad Abu-Remaileh, Abed Khalaileh, Eli Pikarsky, Rami I. Aqeilan

**Affiliations:** 1 The Lautenberg Center for General and Tumor Immunology, Department of Immunology and Cancer Research-IMRIC, Hebrew University-Hadassah Medical School, Jerusalem, Israel; 2 Department of Surgery, Hebrew University-Hadassah Medical, Jerusalem, Israel; 3 Department of Cancer Biology and Genetics, Wexner Medical Center, The Ohio State University, Columbus, OH USA

## Abstract

Liver cancer is one of the most lethal malignancies with very poor prognosis once diagnosed. The most common form of liver cancer is hepatocellular carcinoma (HCC). The WW domain-containing oxidoreductase (*WWOX*) is a large gene that is often perturbed in a wide variety of tumors, including HCC. WWOX has been shown to act as a tumor suppressor modulating cellular metabolism via regulating hypoxia-inducible factor 1α (HIF-1α) levels and function. Given that WWOX is commonly inactivated in HCC, we set to determine whether specific targeted deletion of murine *Wwox* affects liver biology and HCC development. WWOX liver-specific knockout mice (*Wwox*^*ΔHep*^) showed more potent liver regeneration potential and enhanced proliferation as compared with their control littermates. Moreover, WWOX deficiency in hepatocytes combined with diethylnitrosamine treatment increased the tumor burden, which was associated with increased HIF1α levels and target gene transactivation. Inhibition of HIF1α by systemic treatment with digoxin significantly delayed HCC formation. Our work suggests that WWOX inactivation has a central role in promoting HCC through rewiring of cellular metabolism and modulating proliferation.

## Introduction

Hepatocellular carcinoma (HCC) is the most common type of primary liver cancer, representing the fifth type of commonly diagnosed cancer worldwide and third mortality cause among other cancer malignances^1^. HCC prevalence has been dramatically increasing in the last decay because of the expansion of HCC risk factors, including hepatitis infection and obesity^2^. Therapeutic options are limited and survival after diagnosis is still poor leading to high mortality. Therefore, better understanding of the molecular basis of HCC is urgently needed.

WW domain-containing oxidoreducatase (*WWOX*) gene resides in one of the most common fragile sites known as FRA16D, a region that is altered in many types of cancer^3–5^. Frequent homozygous deletions were reported at this region in aflatoxin B1 exposed HCC^6^, suggesting that it might harbor a tumor suppressor. In particular, WWOX expression is absent or reduced in most of the derived liver cancer cell lines^7^. The gene encodes a 46 kDa protein comprising of two N-terminal WW domains, known to mediate protein–protein interactions and a short-chain dehydrogenase/reductase domain whose specific function is unknown yet^8–10^. Moreover, WWOX was suggested as a modulator of β-catenin protein activity in some HCC cells lines^11,12^. In addition to its genomic re-arrangement and hypermethylation of its regulatory region, WWOX is inactivated by other proteins or microRNAs in HCC cell lines^13–15^. However, no direct *in vivo* evidence linking WWOX tumor suppressor function with HCC development is known so far.

WWOX is commonly reported as a tumor suppressor not only owing to its common loss in many human malignancies but also due to its anti-tumorigenic effect when overexpressed and susceptibility of tumor formation in *Wwox*-mutant mice^8,16–19^. *Wwox* null mice die by the age of 3–4 weeks owing to severe metabolic disorders, mainly lethal hypoglycemia^20^ precluding studying implications of WWOX loss in adult mice. To overcome this limitation, we have recently generated a conditional mouse model in which somatic deletion of *Wwox* is achieved using a specific Cre recombinase^21^, mimicking the alterations frequently observed in human cancers and allowing study of human cancer intervention, development and progression, and assessing therapeutic strategies^22,23^. In this study, we utilized a Cre recombinase driven by the promoter of albumin (*Alb-Cre*), which results in somatic deletion of WWOX in hepatocytes; *Wwox*^*ΔHep*^ mice. Interestingly, Iatan et al. have recently demonstrated that WWOX deletion in a murine mouse model modulates levels of lipoproteins, however, these mice did not spontaneously develop HCC^24^. One of the most widely used and accepted models for HCC development in animal models is the use of N-nitrosodiethylamine (DEN), a known carcinogen, which alkylates DNA bases^25^ and results in HCC tumor formation in a defined kinetic manner^23^.

WWOX anti-tumorigenic functions have been shown to affect genome integrity^26–28^, apoptosis^9,29–31^, cell growth and extracellular matrix signaling^32^, and glucose metabolism^33,34^. WWOX, through its first WW domain, interacts with wide variety of proteins regulating their functions and affecting cellular outcome^9,35^. Previously, WWOX have been shown to physically interact with hypoxia-inducible factor 1α (HIF-1α) and inhibit its activity^33^. Ablation of HIF1α expression in RAS-transformed *Wwox*-deficient mouse embryonic fibroblasts significantly reduced tumourigenicity^33^, suggesting that HIF1α mediates the tumorigenic phenotype of *Wwox*-depelted cells. Nevertheless, the functional association of WWOX-HIF1α has not been demonstrated in a cancer mouse model. Here, we studied the effect of treating *Wwox*^*ΔHep*^ mice with DEN and followed HCC development and progression. We demonstrated that WWOX dysregulation accelerates HCC development through control of HIF1α and other master proliferation gene networks implicated in hepatocarcinogenesis. We also demonstrate that WWOX ablation modulates liver regeneration.

## Results

### WWOX expression is commonly lost in liver cancer

**Fig. 1:**
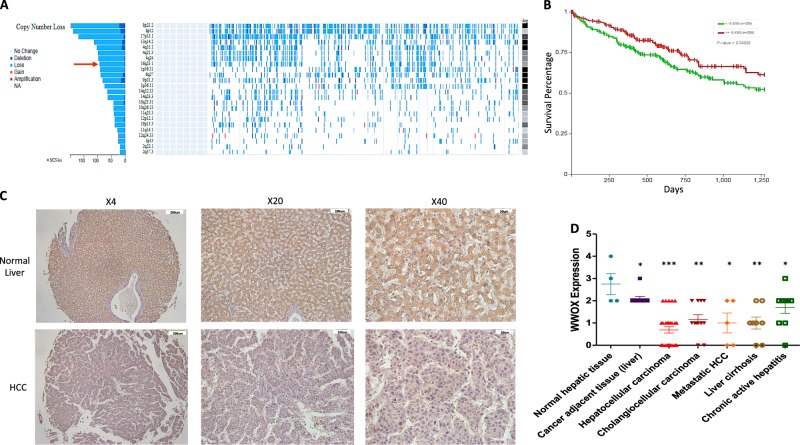
Loss of WWOX expression in liver cancer. **a** TCGA data analysis showing that *WWOX* genomic region spanning long arm of chromosome 16 at 16q23.1 (red arrow) is frequently lost in HCC patients (*n* = 438 cases) with single copy number alteration (SCNA) equal to 96.84%. **b** Prognostic value of *WWOX* mRNA in HCC patients (*n* = 438) showing that patients with low expression had poorer survival compared with those with high *WWOX* expression. *P* value < 0.05. **c** Representative images of a liver cancer tissue microarray (TMA) (LV8011, US Biomax) showing immunohistochemical staining of WWOX in normal and HCC samples. **d** Quantification of WWOX protein levels from **c**. * indicates *P* value < 0.05, ** *P* value < 0.01, *** P value < 0.001. Error bars indicate ± SEM

Given that WWOX expression is altered in many human malignancies^36^, we set to examine its genomic and expression status using different available tools and resources. Analysis of 438 liver cancer samples in the TCGA database using fire browser (www.firebrowse.org) revealed that the *WWOX* locus, spanning chromosomal region of 16q23.1, is one of the most significant regions harboring copy number loss in HCC patients (Fig. 1a). Using Xena browser (www.xenabrowser.net) we next evaluated the prognostic value of WWOX expression in HCC and found that cases harboring reduced *WWOX* mRNA levels tend to present with worse survival outcome compared to those having high WWOX expression (Fig. 1b). Consistent with these observations we further found that WWOX levels are absent or reduced in different liver pathologies, particularly in HCC, as assessed by immunohistochemical staining of a commercial tissue microarray (Fig. 1c, d). Notably, a tendency of reduced WWOX expression was observed in cancer adjacent liver tissue (*P* *<* 0.05*)*, suggesting that loss of WWOX could be an early event in liver carcinogenesis.

### Hepatocyte-specific WWOX ablation accelerates HCC development

**Fig. 2:**
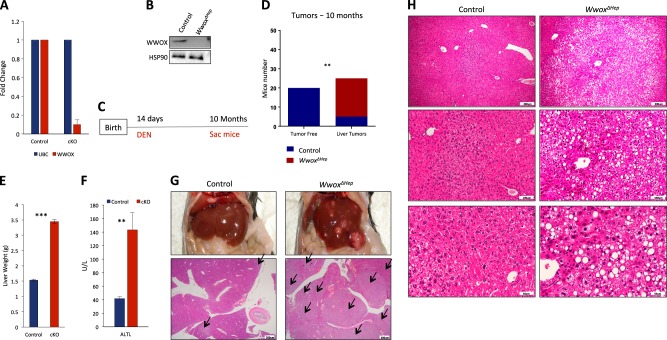
Hepatocyte-specific WWOX ablation accelerates HCC development. **a** Validation of WWOX depletion in *Wwox*^*ΔHep*^ (cKO: conditional knockout) mice using qRT-PCR; *n* = 3 for each mouse group. Error bars indicate ± SEM. **b** Validation of WWOX protein ablation by immunoblotting using WWOX antibody; HSP90 was used as loading control. **c** DEN treatment plan: DEN was IP injected to control and *Wwox*^*ΔHep*^ mice at the age of 14 days and mice were analyzed at the age of 10 months. **d** *χ*^2^ analysis of macroscopic tumor incidence in control mice versus *Wwox*^*ΔHep*^ mice treated with DEN. **e** Tumor load analysis represented by liver weight of control mice (n = 15) versus *Wwox*^*ΔHep*^ cKO mice (*n* = 14) after 10 months of DEN treatment. **f** Serum ALTL levels of control mice (n = 6) versus *Wwox*^*ΔHep*^ mice (*n* = 6) after 10 months of DEN injection. **g** Representative pictures of control and *Wwox*^*ΔHep*^ livers of two male littermates (upper). Histological images showing liver sections of 10 months DEN-treated control and *Wwox*^*ΔHep*^ mice (lower panel) **h** Histological images (**×** 10, **×** 20, and **×** 40) of 10 months DEN-treated mice. * indicates *P* value < 0.05, ** *P* value < 0.01, *** *P* value < 0.001. Error bars indicate ± SEM

The fact that WWOX expression is reduced in early liver cancer lesions suggests that WWOX may have a role in suppressing liver carcinogenesis. To determine whether WWOX loss could contribute to HCC development or progression, we generated a hepatocyte-specific *Wwox* knockout mouse model and followed liver tumor formation. *Wwox*-floxed mice (*Wwoxf/f*)^21^ were bred with *Albumin-Cre* transgenic mice^22^ to generate *Wwoxf/f;Albumin-Cre* (*Wwox*^*ΔHep*^) mice on the C57Bl6/J;SVJ129 mixed genetic background. Successful ablation of WWOX was validated using quantitative real-time (qRT)-PCR and western blot analyses (Fig. 2a, b). Follow up of *Wwox*^*ΔHep*^ and controlled littermate mice for up to 2-years did not reveal spontaneous tumor development, consistent with previous observations^24^. We therefore decided to examine the effect of WWOX ablation upon N-nitrosodiethylamine (DEN) treatment, a widely used chemical carcinogen for studying liver carcinogenesis^22^. Cohorts of *Wwox*^*ΔHep*^ mice and control littermates were intraperitoneally (IP) injected with 5 mg/Kg single injection of DEN at the age of 14 days and monitored for HCC development as a function of time (Fig. 2c). DEN treatment using this protocol is known to lead to 85% HCC development in C57BL6/J strain by the age ranging from 10 to 18 months^37^, whereas 129SVJ strain develop HCC later^38^. As shown in Fig. 2d, DEN-treated *Wwox*^*ΔHep*^ mice developed significantly higher incidence of HCC as compared with control mice (*P* = 0.0025). By the age of 10 months, the penetrance of tumor development was 100% in *Wwox*^*ΔHep*^ mice whereas just 20% of the control littermates’ mice developed macroscopic tumors (Fig. 2d). Furthermore, tumor load, as assessed by liver weight relative to body weight, was significantly higher in *Wwox*^*ΔHep*^ mice as compared with control mice (Fig. 2e). Interestingly, WWOX mRNA and protein was decreased in the tumors of control mice as revealed by qRT-PCR (Figure S1A) and immunohistochemistry (Figure S1B). In addition, *Wwox*^*ΔHep*^ mice had higher levels of serum alanine transaminase, indicating liver dysfunction as a result of tumor formation (Fig. 2f). Histological characterization of liver tissues revealed aggressive, poorly differentiated and highly proliferative tumors resembling HCC (Fig. 2g, h). Altogether, these findings indicate that WWOX loss accelerates HCC development.

### Hepatocyte-specific WWOX ablation is associated with increased proliferation

**Fig. 3:**
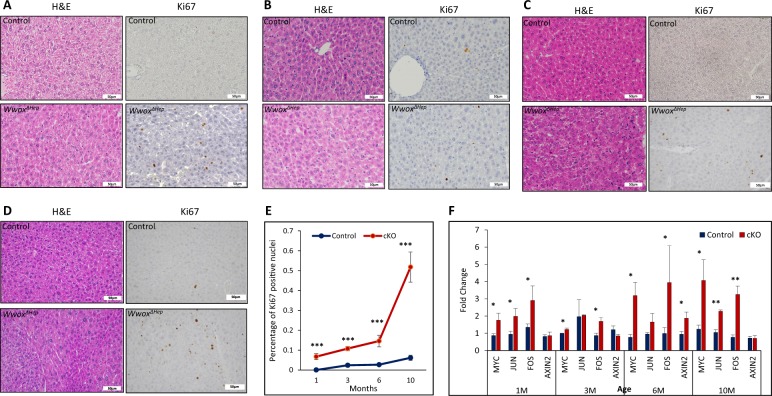
Hepatocyte-specific WWOX ablation is associated with increased proliferation. Histological images (H&E staining and Ki67 immunohistochemical staining at **×** 40) of DEN-treated control and *Wwox*^*ΔHep*^ liver sections at the age of 1 **a**, 3 **b**, 6 **c**, and 10 months **d**. **e** Quantification of positive Ki67 nuclei in 1, 3, 6, and 10 months old DEN-treated control and *Wwox*^*ΔHep*^ mice liver. (*n* = 5 for each group). **f** mRNA expression levels of proliferation genes in DEN-treated control and *Wwox*^*ΔHep*^ mice at the age of 1, 3, 6, and 10 months (*n* = 3 for each group). * indicates *P* value < 0.05, ** *P* value < 0.01, *** *P* value < 0.001. Error bars indicate ± SEM

Our findings so far suggest that hepatocyte-specific WWOX deletion promoted HCC development. On one hand, WWOX overexpression was previously shown to induce apoptosis and suppress proliferation, whereas its loss is associated with enhanced survival in a hepatoma cell line^39^. On the other hand, DEN treatment is known to induce acute hepatic injury followed by compensatory proliferation^40^. We therefore set to determine whether the phenotype observed in DEN-treated *Wwox*^*ΔHep*^ mice is a consequence of impaired proliferation control. To this end, we analyzed liver tissues of DEN-treated mice at different time points starting from 1 to 10 months. Hematoxylin and Eosin (H&E) evaluation revealed that *Wwox*^*ΔHep*^ livers display hepatocyte morphological changes starting from 6-month post DEN treatment, which progressed to HCC at the age of 10 months, as shown earlier (Fig. 3a, d). No histological abnormalities were observed in the groups of 1 and 3-month post DEN treatment of *Wwox*^*ΔHep*^ mice nor in the control mice. To further support our findings, we immunostained for Ki67, a surrogate marker of proliferation. Digital quantification of Ki67-positive nuclei demonstrated that *Wwox*^*ΔHep*^ liver sections had significantly higher number of Ki67-positive cells in the groups of 1, 3, 6, and 10 months *Wwox*^*ΔHep*^ mice relative to the control littermate groups (Fig. 3e). In addition, qRT-PCR analysis of proliferative genes implicated in liver cancer, including c-*Myc, c-Jun, c-Fos, and Axin*, display higher levels in the *Wwox*^*ΔHep*^ mouse groups from as early as 1-month post DEN treatment (Fig. 3f). Although, DEN-free *Wwox*^*ΔHep*^ mice display no tumorigenic phenotype, levels of the previous mentioned proliferative genes were also elevated (Figure S2A). Consistent with these results, a significant increase in *CTGF* levels, one of the main effectors of the Hippo pathway^41^, was also noted in livers of *Wwox*^*ΔHep*^ mice with a slight increase in liver weight in the pre-tumorigenic phase (Figure S2B and S2C). These findings suggest that WWOX has a critical role in inhibiting proliferation of DEN-treated hepatocytes, whereas its inactivation leads to increased proliferation contributing to tumor development.

### Enhanced expression of glycolytic genes in livers of DEN-*Wwox*^*ΔHep*^ mice

**Fig. 4:**
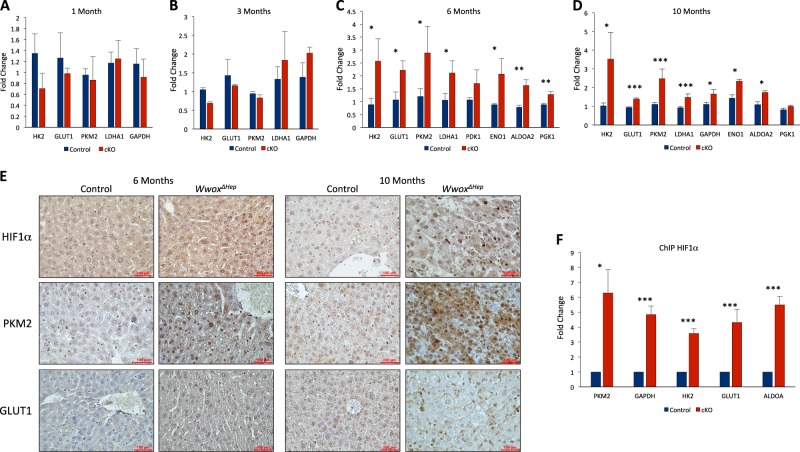
Enhanced expression of glycolytic genes in livers of DEN-*Wwox*^*ΔHep*^ mice. **a** mRNA expression levels of HIF1α glycolytic target genes in DEN-treated control and *Wwox*^*ΔHep*^ (cKO) mice at the age of 1 **a**, 3 **b**, 6 **c** and 10 **d** months (*n* = 3 for each group). **e** Immunohistochemical staining of HIF1α, PKM2, and GLUT1 in 6 and 10 months of DEN-treated *Wwox*^*ΔHep*^ and control liver tissues. Images were taken at **×** 40 magnification (Bar = 100μm). **f** ChIP experiment with HIF1α antibody on DEN-treated control and *Wwox*^*ΔHep*^ (cKO) mice followed by qRT-PCR analysis of HIF1α target genes. * *P* value < 0.05, ** *P* value < 0.01, *** *P* value < 0.001. Error bars indicate ± SEM

Recently, it was shown that WWOX loss is associated with Warburg effect and impaired mitochondrial respiration^33,42,43^. In mammalian cells, WWOX ablation is inversely correlated with increased levels and activity of hypoxia-inducible factor 1α (HIF1α-enhancing cell transformation and tumor growth^33,44^. In particular, *Wwox*-deficient cells were shown to display higher levels of glycolytic genes known to be regulated by HIF1α. Given that HIF1α has known roles in HCC development^45^, we next set to determine whether WWOX-targeted loss in the different DEN-treated *Wwox*^*ΔHep*^ and control mice is associated with impaired HIF1α function. Analysis of HIF1α glycolytic target genes mRNA, including *Hk2*, *Pkm2*, *Glut1* and others, demonstrated no change at 1 and 3-months post treatment (Fig. 4a, b). Intriguingly, a significant upregulation of these transcripts was noted in 6 and 10 months DEN-treated *Wwox*^*ΔHep*^ mice compared with control mice (Fig. 4c, d). Interestingly, DEN-free *Wwox*^*ΔHep*^ mice on 6 months age displayed a tendency of HIF1α glycolytic target genes upregulation (Figure S3A). Moreover, nuclear HIF1α protein levels exhibited moderate and prominent increase at 6 and 10 months in DEN-treated *Wwox*^*ΔHep*^ mice, respectively (Fig. 4f). Likewise, levels of PKM2 and membranous GLUT1 protein were higher in 6 and 10 months DEN-treated *Wwox*^*ΔHep*^ mice (Fig. 4e).

Our recent studies have shown suppression of HIF1α transactivation function by WWOX^29^. Therefore, we next determined whether WWOX loss modulates HIF1α recruitment to promoters of these glycolytic genes. To address this, we performed a chromatin immunoprecipitation (ChIP) experiment using anti-HIF1α antibody on genomic DNA isolated from liver tissues of 10 months DEN-treated *Wwox*^*ΔHep*^ and control littermate mice. QRT-PCR analysis revealed HIF1α enrichment on promoters of target genes, including *Pkm2*, *Hk2*, *Glut1*, *Aldh*, and *Gapdh*, in *Wwox*^*ΔHep*^ livers (Fig. 4f). Altogether, these results imply that WWOX loss leads to HIF1α enhanced activity and, likely, reprogramming of HCC cells to glucose metabolism and Warburg effect.

### Inhibition of HIF1α rescued the effect of WWOX loss

**Fig. 5:**
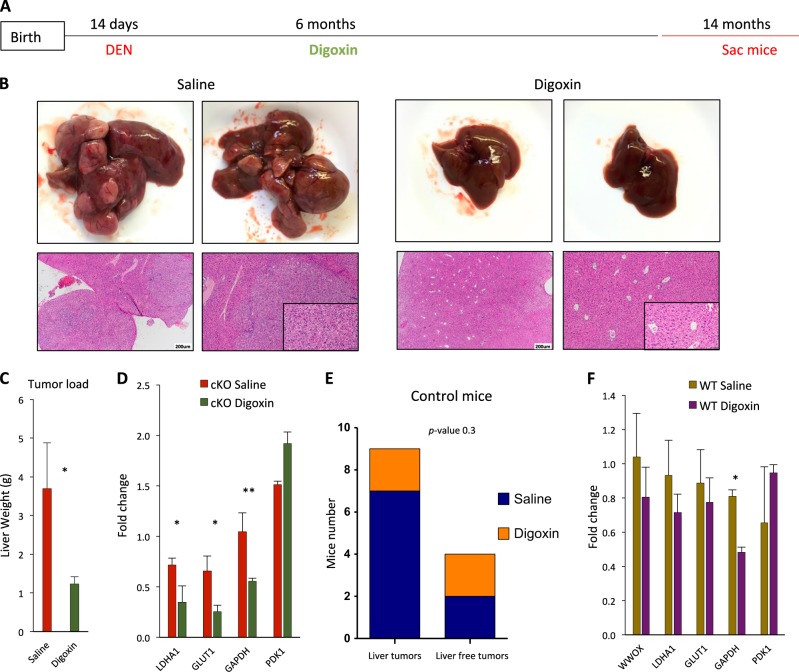
Inhibition of HIF1α rescued the effect of WWOX loss. **a** Digoxin experiment plan, DEN was IP injected to control and *Wwox*^*ΔHep*^ mice at the age of 14 days. Six-month later mice were started to be treated with digoxin (1 mg/kg) or vehicle control (saline) 3-times a week for an additional 8-months. **b** Representative images of saline-treated *Wwox*^*ΔHep*^ (cKO) mice versus digoxin-treated *Wwox*^*ΔHep*^ (cKO) mice livers and liver sections **c** Tumor load of saline-treated *Wwox*^*ΔHep*^ mice versus digoxin-treated *Wwox*^*ΔHep*^ mice livers as assessed by liver weight at the age of 14 months **d** mRNA expression levels of HIF1α glycolytic target genes in saline-treated *Wwox*^*ΔHep*^ (cKO) mice versus digoxin-treated *Wwox*^*ΔHep*^ (cKO) mice at the age of 14 months, (*n* = 3 for each group). **e** *Χ*^2^ analysis of macroscopic tumor incidence in saline-treated control mice versus digoxin-treated control mice at the age of 14 months. **f** mRNA expression levels of HIF1α glycolytic target genes in saline-treated control mice versus digoxin-treated control mice at the age of 14 months, (*n* = 3 for each group). * *P* value < 0.05, ** *P* value < 0.01, *** *P* value < 0.001. Error bars indicate ± SEM

We have previously shown that digoxin, an inhibitor of HIF1α, rescued the effect of *Wwox*-deficient mice displaying hypoglycemia and elevated levels of glycolytic genes^33^. Digoxin is a cardiac glycoside, which has been shown to inhibit HIF1α transcriptional activity *in vitro*^46^ and suppress tumor growth *in vivo*^47^. We therefore set to investigate whether digoxin treatment of DEN-*Wwox*^*ΔHep*^ mice could reduce the incidence of HCC development. DEN-*Wwox*^*ΔHep*^ and control mice received digoxin (1 mg/kg) or vehicle control (saline) through intraperitoneal injections (3-times/week) for 32 weeks starting at 6 months post DEN treatment (Fig. 5a). Analysis of mice 8-months post digoxin treatment revealed marked suppression of liver tumor growth with more prominent effect in *Wwox*^*ΔHep*^ mice compared with saline-treated *Wwox*^*ΔHep*^ mice (Fig. 5b, c). In contrast, no significant difference was observed between digoxin and saline-treated DEN control mice (Fig. 5e). Consistent with these results, digoxin-treated DEN-*Wwox*^*ΔHep*^ mice showed downregulations of HIF1α targets, whereas no significant changes were observed in digoxin-treated DEN control mice (Fig. 5d, f). We conclude that WWOX loss exerts critical regulation on HIF1α function mediating rewiring of glucose metabolism and contributing to enhanced proliferation.

### High-fat diet (HFD) increased HCC incidence in *Wwox*^*ΔHep*^ mice

**Fig. 6:**
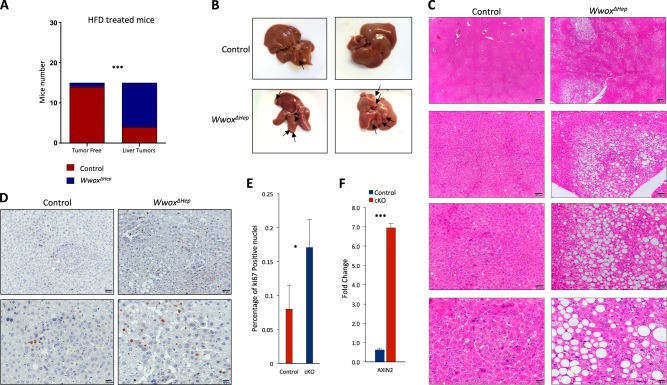
High-fat diet (HFD) increases HCC incidence in DEN-treated *Wwox*^*ΔHep*^ mice. **a** *Χ*^2^analysis of HCC incidence in DEN-treated control and *Wwox*^*ΔHep*^ mice (at age of 14 days) and fed with HFD from the age of 8 weeks. **b** Representative pictures of control and *Wwox*^*ΔHep*^ (cKO) livers fed with HFD at the age of 7 months. Arrows indicate macroscopic tumors. **c** Histological images (× 4, × 10, × 2,0 and ×40) of 7 months DEN-treated control and *Wwox*^*ΔHep*^ livers fed with HFD. **d** Histological images (Ki67 immunohistochemical staining) of mice from **c**. **e** Quantification of positive Ki67 nuclei of mice from **d**. **f** mRNA expression of *Axin2* (control, *n* = 3; cKO, n = 3). * *P* value < 0.05, ** *P* value < 0.01, *** *P* value < 0.001. Error bars indicate ± SEM

Obesity is known to be a major risk factor for HCC development. Indeed, combined HFD and DEN treatment have been previously shown to strongly enhance HCC development when compared with DEN mice on normal diet^48^. Driven by our previous observations, we decided to investigate the effect of HFD on HCC development in DEN-*Wwox*^*ΔHep*^ mice. DEN-treated cohorts of male *Wwox*^*ΔHep*^ and control mice were placed on HFD, in which 60% of calories were fat-derived, for 30 weeks. Tumors of DEN-treated *Wwox*^*ΔHep*^ HFD mice appeared 3-months earlier compared with normal chow-diet mice (Fig. 2), consistent with previous reported data showing acceleration of HCC formation in mice fed with HFD^48^. No difference of mice weight between the two groups of HFD-*Wwox*^*ΔHep*^ and control mice was noted (Figure S4A). Nevertheless, the number of tumors in *Wwox*^*ΔHep*^ mice was significantly higher (Fig. 6a, b) and was accompanied with higher levels of serum ALT (Figure S4B) and hepatosteatosis (Fig. 6c). Consistent with anti-proliferative role of WWOX that we showed previously (Fig. 3), DEN-treated *Wwox*^*ΔHep*^ mice fed with HFD displayed increased proliferation (Fig. 6d, e). Persistent with a prominent role of β-catenin pathway in HCC, we observed significant upregulation of *Axin2* transcript in DEN-treated *Wwox*^*ΔHep*^ HFD mice (Fig. 6f). At a later stage, there was also an upregulation of HIF1α-target genes (Figure S4C). Altogether, our findings suggest that combined loss of WWOX and HFD intake accelerate HCC formation mediated by enhanced proliferation.

### *Wwox* ablation is associated with increased proliferation upon partial hepatectomy

**Fig. 7:**
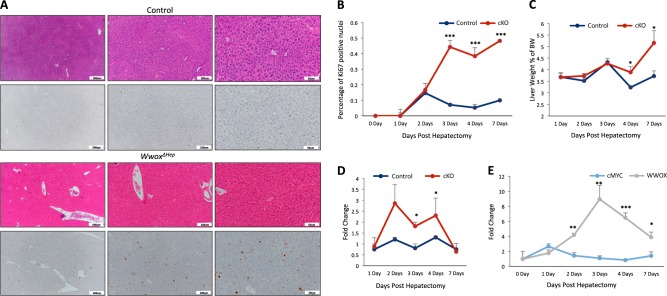
*Wwox* ablation is associated with increased proliferation upon partial hepatectomy. **a** Histological images (H&E staining and Ki67 immunohistochemical staining) of control and *Wwox*^*ΔHep*^ mice liver 3-days post 50% partial hepatectomy (× 10, × 20, and × 40). **b** Quantification of positive Ki67 nuclei in 5 months old control (WT) and *Wwox*^*ΔHep*^ mice liver 1, 2, 3, 4, and 7 days post hepatectomy (*n* = 5 for each group). **c** Liver weight to body weight ratio of 1, 2, 3, 4, and 7 days post hepatectomy (*n* = 5 for each group). **d** mRNA expression levels of *c-Myc* in control and *Wwox*^*ΔHep*^ mice liver 1, 2, 3, 4, and 7 days post hepatectomy. **e** mRNA expression levels of *Wwox* in control and *Wwox*^*ΔHep*^ mice liver 1, 2, 3, 4, and 7 days post hepatectomy (*n* = 4 for each group). * *P* value < 0.05, ** *P* value < 0.01, *** *P* value < 0.001. Error bars indicate ± SEM

The preceding observations demonstrated that WWOX inactivation coupled with DEN treatment and HFD intake leads to enhanced proliferation and tumor formation. We next asked whether WWOX deletion affects hepatic cell proliferation upon partial hepatectomy, a process known to acutely induce hepatocyte proliferation to achieve liver regeneration. *Wwox*^*ΔHep*^ mice and their littermate controls were subjected to 30% hepatectomy and were analyzed 1, 2, 3, 4, and 7 days later for Ki67 immunohistochemical staining. As expected, an increase in the proliferation index was observed in *Wwox*^*ΔHep*^ mice compared with control mice (Fig. 7a, b). Intriguingly, whereas the proliferation index was reduced 4 and 7 days post hepatectomy in control mice, it continued rising in *Wwox*^*ΔHep*^ mice. Increased proliferation was associated with increased ratio of liver to body weight on day 7 post hepatectomy (Fig. 7c). Moreover, qRT-PCR analysis of proliferative gene implicated in liver regeneration, including c-*Myc*, displayed higher levels in *Wwox*^*ΔHep*^ mice group on day 2, 3, and 4 as compared with control mice (Fig. 7d). Interestingly, *Wwox* and c-*Myc* transcripts showed a trend of inverse pattern of expression in *Wwox-*control mice (Fig. 7e) suggesting that WWOX might negatively modulate c-Myc. These results are consistent with WWOX function to inhibit hepatocyte proliferation after liver regeneration.

## Discussion

We report here that WWOX loss is a common event in HCC and that its dysregulation synergizes with DEN treatment and HFD to accelerate HCC development through regulation of proliferation genes. At the molecular level, WWOX, known to act as an adapter protein through interaction via its WW1 domain, functionally associates with HIF1α and probably other transcription factors involved in proliferation, leading to inhibition of their transactivation function. For example, when WWOX is lost, HIF1α recruitment and function on its target genes are enhanced leading to increased expression of glycolytic genes and proliferation rate. We further demonstrate that pharmacological inhibition of HIF1α reduces HCC burden in DEN-treated liver-specific WWOX-deficient mice. Finally, we show that WWOX loss increases liver regeneration after hepatectomy possibly via eliminating the termination phase of liver regeneration. These findings underscore the significant role of WWOX in rewiring metabolic changes in liver cells contributing to liver cancer.

Several studies have suggested that the *WWOX* gene functions as a tumor suppressor in liver cancer^3,4,7^. Early evidence has revealed the existence of frequent homozygous deletions at chromosome 16q23, where WWOX resides, in aflatoxin B1 exposed HCC^6^. In another study, expression of both *WWOX* and *FHIT*, another tumor suppressor located in a fragile site, appeared to be correlated and down-regulated in liver tissues in a carcinogen-specific manner^49^. A subsequent study has examined the status of the *WWOX* gene in human HCC cell lines and found its recurrent alterations further implicating WWOX in hepatocarcinogenesis^7^. In our study, we further delineate the role of WWOX as a tumor suppressor in liver cancer. First, we showed that WWOX expression is reduced or absent in large cohorts of human liver pathologies, including HCC. Furthermore, WWOX low expression in HCC is correlated with decreased survival suggesting that WWOX expression has a prognostic value in HCC. Second, we provide the first *in vivo* evidence that WWOX loss contributes to HCC development, in two different models of murine HCC. Our data show that DEN treatment alone or in combination with HFD clearly have an advantage for HCC development upon WWOX loss. Both tumor incidence and multiplicity (load) were higher in *Wwox*^*ΔHep*^ mice implying the importance of WWOX alteration for HCC development and progression. Our findings also suggest that WWOX loss is an important contributor for HCC promotion as earlier hits are required for HCC initiation. WWOX expression could be altered by environmental cues^6,49^ or in consequence of microRNA dysregulation^15^ or even as a result of genetic variations in WWOX^50^. If these alterations were combined with western HFD then our findings indicate that HCC risk increases.

We also showed that WWOX anti-proliferative activity is mediated by suppression of the proto-oncogenes c-*Myc*, c-*Fos*, and c-*Jun*. c-*Myc* is known as an oncogene that promotes HCC in human^51^ and murine HCC models^52,53^. We found that c-*Myc* is upregulated in *Wwox*^*ΔHep*^ mice on 1, 3, 6, and 10 months continually, raising the point that WWOX partially suppresses HCC through continuous suppression of c-Myc. Previous reports have shown that WWOX suppresses the AP-1 transcriptional activity through regulating c-Jun localization^54^, consistent with our findings showing enhanced expression of c-Jun and c-Fos in WWOX-depleted cells. It is also likely that WWOX loss releases its inhibitory effect on a plethora of other proto-oncogenes as have been previously demonstrated^9,16,55^, contributing to the observed aggressive HCC phenotype. Although WWOX has been shown as an effector of the Wnt/β-catenin pathway *in vitro*, through interaction with DVL^15,35,56^, our results do not support such an effect in DEN-mediated HCC development. On the other hand, when DEN treatment was combined with HFD, WWOX loss resulted in significant increase in Axin levels, suggesting that under these conditions WWOX plays a more important role in regulating the Wnt/β-catenin pathway. It is therefore possible that other cellular pathways are also involved as WWOX function has been shown to include plethora of effectors^57^.

Our data further present a critical metabolic and anti-proliferative role of the tumor suppressor *WWOX* in suppressing HCC through regulation of HIF1α function. The fact that HIF1α protein levels and its target genes were elevated in the pre-tumor stage (6 months; Fig. 4e) implies that these metabolic changes fuel and drive HCC development in DEN-treated *Wwox*^*ΔHep*^ mice. HIF1α is significantly elevated in human HCC samples and associated with bad prognosis^58,59^. Moreover, HIF1α hepatocytes-specific overexpression in a murine model increases HCC-promoting M2 macrophages^60^, whereas HIF1α liver-specific knockout sensitizes hepatoma cells to etoposide treatment^61^. WWOX loss is associated with elevated HIF1α target genes starting from the age of 6 months. In addition, we show that HIF1α is enriched on its targets promoters in hepatocytes isolated from *Wwox*^*ΔHep*^ mice.

Targeting tumor suppressor genes is a major challenge. Novel approaches, including synthetic lethality and collateral vulnerability have been proposed to come over this limitation. We therefore, assessed whether targeting the proliferative/survival signals in WWOX-deficient cancer cells could help inhibit tumor progression. Several attempts to target HIF1α in HCC were reported via inhibition of its expression^62^, dimerization^63^, or activity^64^. In our model, we were able to rescue, at least partially, the tumor phenotype using an HIF1α inhibitor (digoxin); at the age of 14 months, DEN-*Wwox*^*ΔHep*^ mice did not show macroscopic tumors and had lower expression of glycolytic genes driven by HIF1α. These results are consistent with our previous data showing that WWOX inhibits aerobic glycolysis^42–44,65,66^. Rewiring of glucose metabolism in *Wwox*^*ΔHep*^ mice provides the needed building blocks for cell division and proliferation^67^ and hence these mice could have more proliferation compared with control mice. Future studies shall include more specific inhibitors of HIF1a and probably Myc to inhibit WWOX-mediated HCC development.

WWOX anti-proliferative function in HCC might also involve modulation of fatty acid/lipid metabolism. In fact, WWOX genetic variants in human patients^68^ and *Wwox* knockout mouse models^24^ display decreased serum HDL-C. Moreover, microarray analyses of *Wwox* liver-specific knockout mice revealed an increase in plasma triglycerides and altered lipid metabolic pathways suggesting that WWOX disruption indeed alters cellular lipid homeostasis in the liver. Whether these WWOX effects can also contribute to HCC development is unknown.

Our findings also present a vital role of WWOX in maintaining healthy liver regeneration. Remarkably, partial hepatectomy in liver-specific *Wwox*-deficient mice resulted in increased proliferation (Ki67) upon liver regeneration, at the later phases of this remarkably controlled homeostatic process. Interestingly, *Wwox* RNA levels increased 2 days post hepatectomy while c-*Myc* levels showed an inverse pattern. c-*Myc* levels are reported to be highest in 12–18 h post heptectomy and then *c-Myc* levels usually drop down^69–71^. Our results might suggest that *WWOX*, an anti-proliferative gene, is induced during the course of liver regeneration to allow shutting down proliferative genes, such as c-*Myc*, and maintaining proper organ size. Interestingly, no difference in HIF1α target genes was observed in liver regeneration of *Wwox*^*ΔHep*^. These observations suggest that WWOX may regulate liver regeneration by HIF1α-independent mechanism.

In conclusion, our study reveals WWOX as a tumor suppressor with critical roles in HCC suppression through maintaining moderate glucose metabolism and inhibiting uncontrolled cell proliferation.

## Materials and Methods

### Mice and related experiments

*Wwox*-floxed (*Wwoxfl/fl*) C57BL6/J;129sv mixed genetic background mice were bred with *Albumin-Cre* transgenic mice to generate *Wwox* conditional knockout in hepatocytes (*Wwox*^*ΔHep*^ mice). Male pups of control and *Wwox*^*ΔHep*^ mice were IP injected with 5 mg/Kg DEN (Sigma Aldrich) at the age of 14 days. Partial hepatectomy (30%) of the liver was done on 4-month-aged males as described^72^. For digoxin treatment, digoxin (1 mg/Kg, Sigma Aldrich) was IP injected into DEN-treated mice starting at age of 6.5 months, 3-times/week, for a total of 8-months. The mice in HFD experiment were fed by 60% kcal fat (Research Diets INC, D12492) for 30 weeks. Tumor load was assessed by liver weight in grams. All experiments involving mice were approved by the Hebrew University Institutional Animal Care and Use Committee.

### RNA extraction and Real-time PCR

Total RNA was prepared using TRI reagent (Sigma Aldrich) following instructions of the manufacturer. One microgram of RNA was used for complementary DNA synthesis using First-Strand cDNA Synthesis kit (Bio-Rad, Hercules, CA). QRT-PCR was performed using Power SYBR Green PCR Master Mix (Applied Biosystems, Foster City, CA). All measurements were performed in triplicate and standardized to the levels of the *Ubc* gene. List of primers used is provided as Supplemental Table 1.

### Histology and immunohistochemistry

Tissues were fixed in 4% neutral buffered-formalin and then paraffin embedded, sectioned, and stained with H&E. Immunohistochemical staining was done as previously described^73^. Immunohistochemical staining of WWOX (polyclonal anti-WWOX antibody, dilution, 1:4000 for 1 h) was done after antigen retrieval with 10 mM citrate buffer (pH 6.0) in a pressure cooker. Detection was done with DAB peroxidase substrate kit (Cat# SK-4100, Vector). Antibodies used were: Ki67 antibody (Cat# MA5-14520, Thermoscientific, dilution 1:200), PKM2 antibody (Cat# 38237, Abcam, dilution 1:50) and HIF1α antibody (Cat# NB100-105, Novus, dilution 1:20).

### ChIP

Hepatocytes were isolated from liver tissues, and solutions were prepared for ChIP analysis according to a standard protocol^74^. 0.8 mg of HIF1α antibody (mouse mAB, Cat # NB100-105, Novous Biological, CO, USA) was used to precipitate HIF1α. Targeted PCR was done using list of primers (Supplemental Table 2).

### Statistics

Results of the experiments were expressed as mean ± standard deviation or standard error of mean. Student’s *t-*test, was used to compare values of test and control samples. *P* < 0.05 indicates significant difference. * *P*-value < 0.05, ** *P*-value < 0.01. For the human data analysis, Single Copy Number Alteration of liver hepatocellular carcinoma (LiHC) TCGA data set (*n* = 434) was analyzed by the website www.firebrowse.org (developed by broad institute of MIT and Harvard). Survival curve of LiHC TCGA data set was analyzed by the web site www.xenabrowser.net (Developed by UCSC).

## Electronic supplementary material

Supplemental Info
