# Author Correction: WWOX controls hepatic HIF1α to suppress hepatocyte proliferation and neoplasia

**DOI:** 10.1038/s41419-018-1158-9

**Published:** 2018-11-23

**Authors:** Muhannad Abu-Remaileh, Abed Khalaileh, Eli Pikarsky, Rami I. Aqeilan

**Affiliations:** 1 The Lautenberg Center for General and Tumor Immunology, Department of Immunology and Cancer Research-IMRIC, Hebrew University-Hadassah Medical School, Jerusalem, Israel; 2 Department of Surgery, Hebrew University-Hadassah Medical, Jerusalem, Israel; 3 Department of Cancer Biology and Genetics, Wexner Medical Center, The Ohio State University, Columbus, OH USA

Correction to: *Cell Death and Disease* (2018); 10.1038/s41419-018-0510-4; published online 03 May 2018.

Since the publication of this Article, the authors reported that duplication had mistakenly occurred in Fig. 3a and d—panels that display H&E.

The corrected Article appears online together with this erratum.

