# Supplementary material for: WWOX controls hepatic HIF1α to suppress hepatocyte proliferation and neoplasia
Source: Cell Death Dis. 2018 May 3;9(5):511. doi: 10.1038/s41419-018-0510-4 (PMC5938702; doi:10.1038/s41419-018-0510-4)
Supplement: Supplementary file 1 — Supplemental Info [file 41419_2018_510_MOESM1_ESM.pdf]

Supplemental Informations

Supplemental Figures Legend

Supplement Figure1.

(A) mRNA expression levels of *Wwox* in the paranchyma and the tumors of 10 months DEN-treated control mice (Paranchyma n=3; Tumor n=3). (B) IHC of tumor bearing 10 months DEN-treated control mouse with WWOX antibody. (P: Paranchyma; T: Tumor). * P-value <0.05, ** P-value < 0.01, *** P-value <0.001. Error bars indicate ± SEM.

Supplement Figure2.

(A) mRNA expression levels of proliferative genes in DEN-free control and $Wwox^{\Delta Hep}$ (cKO) mice (n=4 for each genotype). (B) *CTGF* mRNA expression levels in DEN-treated control and $Wwox^{\Delta Hep}$ mice at the age of 1, 3, 6 and 10 –months, (n=3 for each group). (C) Liver weight of DEN-treated control and $Wwox^{\Delta Hep}$ mice at the age of 1, 3 and 6 months (n=7 for each group). * P-value <0.05, ** P-value < 0.01, *** P-value <0.001. Error bars indicate ± SEM.

Supplement Figure3.

(A) mRNA expression levels of HIF1α glycolytic target genes in DEN-free control and $Wwox^{\Delta Hep}$ (cKO) mice (n=4 for each genotype). * P-value <0.05, ** P-value < 0.01, *** P-value <0.001. Error bars indicate ± SEM.

Supplement Figure4.

(A) Weight increasing with HFD for DEN-treated control and $Wwox^{\Delta Hep}$ mice. (B) Serum ALTL levels of control and $Wwox^{\Delta Hep}$ (cKO) livers fed with HFD at the age of 7 months. (C) mRNA expression of HIF1α glycolytic target genes of DEN-treated control and $Wwox^{\Delta Hep}$ (cKO) livers fed with HFD at the age of 11 months. * P-value <0.05, ** P-value < 0.01. Error bars indicate ± SEM.

Supplemental Tables

Supplement Table 1: List of RT Primers:

Primer	Sequence
mUbc F	CAGCCGTATATCTTCCCA
mUbc R	CTCAGAGGGATGCCAGTA
mWwox F	GGGAGCTGCTACCACTGTCTA
mWwox R	CCTCTCACTGAGTTCCCACA
mGlut1 F	ACCAAAAGCAACGGAGAAGAG
mGlut1 R	GGCATTCCGAAACAGGTAACTC
mLdha1 F	CTGTGTAACTGCGAACTCCAA
mLdha1 R	GTGTGGACTGTACTTGACAATGT
mPdk1 F	AGGATCAGAAACCGGCACAAT
mPdk1 R	GTGCTGGTTGAGTAGCATTCTAA
mHk2 F	GTGTGCTCCGAGTAAGGGTG
mHk2 R	CAGGCATTCGGCAATGTGG
mPkm2 F	GCCGCCTGGACATTGACTC
mPkm2 R	CCATGAGAGAAATTCAGCCGAG
mPgk1 F	TCATGTAGAGGAAG
mPgk1 R	GACATCTCCTAGTTTGGACAGTG
mGapdh F	TGGATTTGGACGCATTGGTC
mGapdh R	TTTGCACTGGTACGTGTTGAT
mEno1 F	TGCGTCCACTGGCATCTAC
mEno1 R	CAGAGCAGGCGCAATAGTTTTA
mAldoa2 F	CGTGTGAATCCCTGCATTGG
mAldoa2 R	CAGCCCCTGGGTAGTTGTC
mAxin2 F	TGACTCTCCTTCCAGATCCCA
mAxin2 R	TGCCCACACTAGGCTGACA
mc-Myc F	ATGCCCCTCAACGTGAACTTC
mc-Myc R	CGCAACATAGGATGGAGAGCA
mJun F	CCTTCTACGACGATGCCCTC
mJun R	GGTTCAAGGTCATGCTCTGTTT
mFos F	CGGGTTTCAACGCCGACTA
mFos R	TTGGCACTAGAGACGGACAGA

Supplement Table 2: List of ChIP primers:

Primer	Sequence
mPKM-Pro-F	AGTGTTCGCAGCTCTTCGTC
mPKM-Pro-R	TGGAGGTTGAAAGGATGCTG
mGAPDH-Pro-F	AGCTACGTGCACCCGTAAAG
mGAPDH-Pro-R	GGTCCAAAGAGAGGGAGGAG
mHK2-Pro-F	ACGTGTGTTCCGTGGCTAGA
mHK2-Pro-R	ACCCGAAGCTGAGCCTGAC
mGlut1-Pro-F	CAGCAGCAAGGTGAGTAACG
mGlut1-Pro-R	GTGAGGAAGCGGGGACTC
mAldoa-Pro-F	CGATAGGAGGCGGCACTG
mAldoa-Pro-R	TGGCTCGAGTCACGTCCT
mGAPDH-Negative-F	CCCCAGGCTATCTCATGTTC
mGAPDH-Negative-R	ATAGCTGATGGCTGCAGGTT
mPGK1-Negative-F	GGGTGCTCTATGGATGTGGT
mPGK1-Negative-R	GAAACATCGTGCTGTGCATT

A

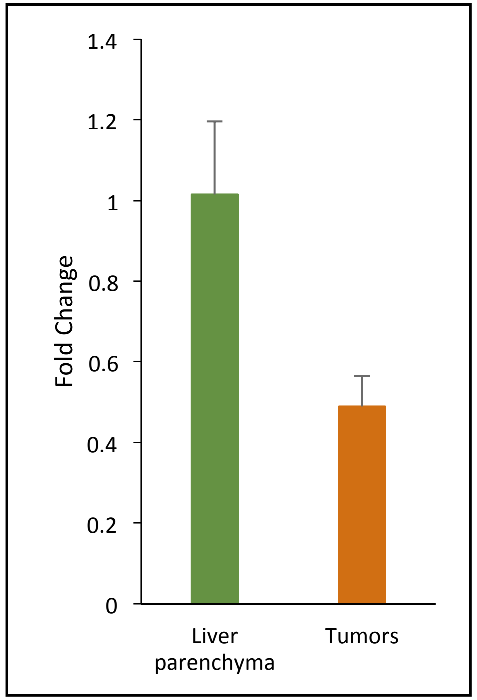

B

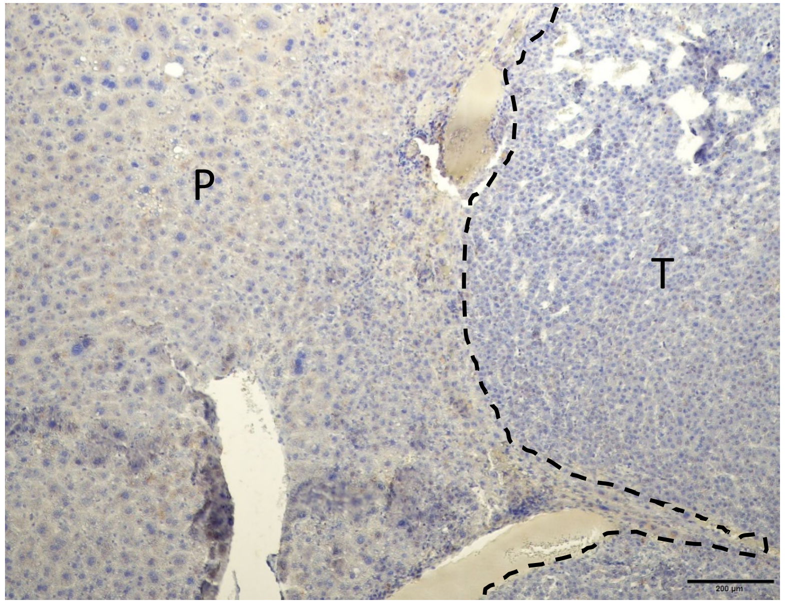

Supplemental Figure 1

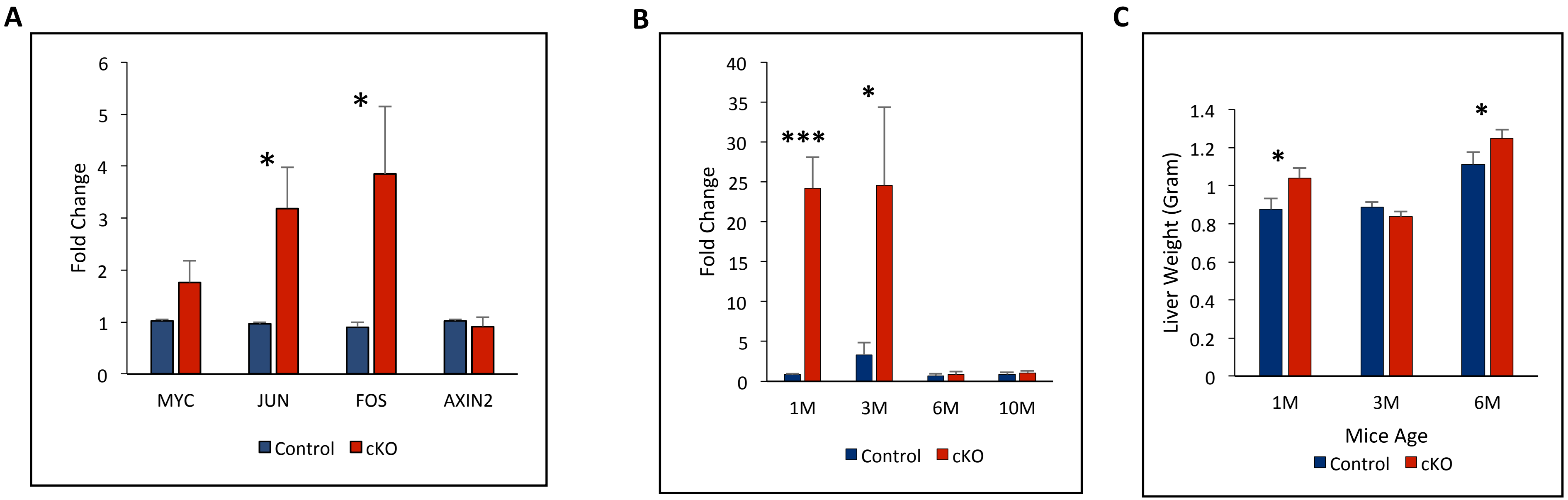

Supplemental Figure 2

A

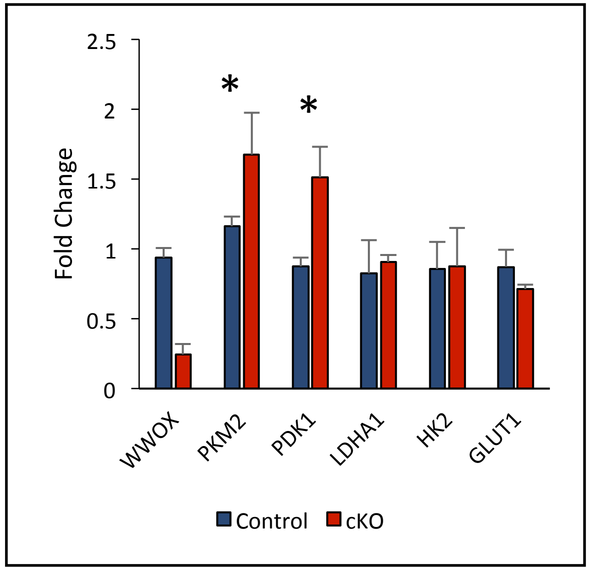

Supplemental Figure 3

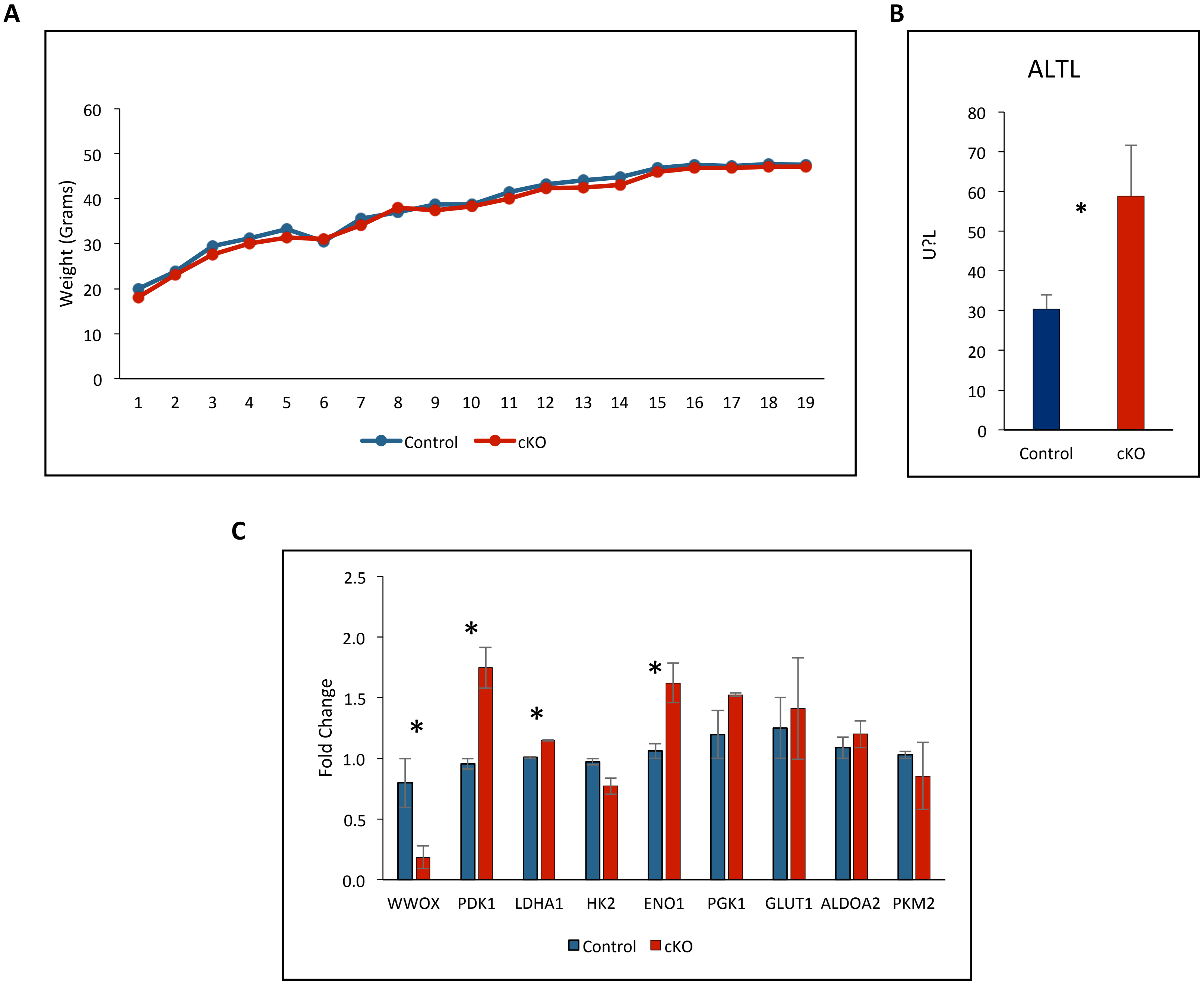

Supplemental Figure 4
